# Male fetal loss in the U.S. following the terrorist attacks of September 11, 2001

**DOI:** 10.1186/1471-2458-10-273

**Published:** 2010-05-25

**Authors:** Tim A Bruckner, Ralph Catalano, Jennifer Ahern

**Affiliations:** 1 Public Health & Planning, Policy and Design, University of California at Irvine, Irvine, CA, USA; 2 School of Public Health, University of California at Berkeley, Berkeley, CA, USA

## Abstract

**Background:**

The secondary sex ratio (i.e., the odds of a male birth) reportedly declines following natural disasters, pollution events, and economic collapse. It remains unclear whether this decline results from an excess of male fetal loss or reduced male conceptions. The literature also does not converge as to whether the terrorist attacks of September 11, 2001 induced "communal bereavement", or the widespread feeling of distress among persons who never met those directly involved in the attacks. We test the communal bereavement hypothesis among gravid women by examining whether male fetal deaths rose above expected levels in the US following September 11, 2001.

**Methods:**

We apply interrupted time-series methods to all fetal deaths at or greater than the 20^th ^week of gestation in the US from 1996 to 2002. Time-series methods control for trends, seasonality, and other forms of autocorrelation that could induce spurious associations.

**Results:**

Results support the hypothesis in that the fetal death sex ratio (i.e., the odds of a male fetal death) increased above its expected value in September 2001. Additional analysis of the secondary sex ratio indirectly supports that the terrorist attacks may have threatened the gestation of male more than female fetuses.

**Conclusions:**

Societal responses to events such as September 11, 2001 do not appear confined only to persons who have ever met the deceased. The fetal death sex ratio in the US population may serve as a sentinel indicator of the degree to which pregnant women react to population stressors.

## Background

The secondary sex ratio (i.e., the odds of a male birth) reportedly falls in populations subjected to natural disasters [1], pollution events [2], and the contraction or collapse of economies [3,4]. Drops in the secondary sex ratio deserve public health attention because the literature indicates that male fetal loss may contribute to this decline [5] Although the biological mechanism remains unclear, male fetuses appear more sensitive than female fetuses to maternal corticosteroids produced after the twentieth week of gestation [6,7]. This elevated stress reactivity apparently jeopardizes the viability of males *in utero*. Consistent with the theory of natural selection, humans may have conserved this male fetal sensitivity to maximize the mother's total yield of grandchildren [8].

One study in California suggests male fetal loss may follow stressful events. The authors report that ambient economic decline precedes an increased risk of a male fetal death [9]. Mechanisms other than male fetal loss, however, may account for the inverse association between environmental stressors and the secondary sex ratio. Ambient stressors may decrease the odds of a male conception by reducing sperm motility or the frequency of coitus [10,11]. Fukuda and colleagues, for instance, found sub-optimal sperm motility among Japanese males after the Kobe earthquake [12].

Distinguishing between the male fetal loss and reduced male conception explanations holds implications for public health. Whereas reduced male conceptions may interest basic researchers, fetal loss induces psychological, if not somatic, morbidity. Mothers who report a stillbirth or spontaneous abortion also exhibit an elevated incidence of subsequent adverse birth outcomes [13,14]. For these reasons, fetal loss remains the object of much clinical intervention.

The terrorist attacks of September 11, 2001 induced widespread social and economic disruption, leading to high levels of stress and anxiety in the United States population [15-17]. The attacks' discrete temporal nature allows us to test whether and when they increased male fetal loss. One report of the fetal death sex ratio (i.e., odds of a male fetal death) after September 11 appears in the literature. In California, the fetal death sex ratio increased one month after the attacks [18]. Reports in California and New York City also indirectly support the male fetal loss explanation, as the secondary sex ratio decreased three and four, but not eight, nine, or ten, months after the attacks [19].

These findings raise the obvious question of whether male fetal loss increased in the remainder of the United States (U.S.) following the terrorist attacks. This test could assist in understanding the extent to which pregnant mothers experienced "communal bereavement" after September 11. Although most research on bereavement concerns individuals with direct relationships to the deceased, work by Catalano and Hartig [20] indicates perinatal sequelae among the broader society without such social ties. The authors posit that a nation's population of pregnant women may experience widespread distress even if they never met the deceased, particularly after events in which institutions such as the state fail to maintain safety and security for its members. We believe that the terrorist attacks of September 11, 2001 meet this condition. Much epidemiologic literature, moreover, reports a nationwide increases in acute mental distress following the attacks of which the overwhelming majority of respondents had no direct relationship to the deceased [15-17]. We also know of no reports that pregnant women in particular sheltered themselves from this distress.

We test the communal bereavement hypothesis that the fetal death sex ratio in the U.S. (less California) rose above its expected level following September 11, 2001. Consistent with theory and earlier empirical research, we, as described below, focus our fetal loss test in September, October, and November 2001 [18]. If results support male fetal loss, we then test the attending hypothesis that the secondary sex ratio in the U.S. (less California and New York City) will fall below its expected value two, three, or four months after September 11, 2001.

## Methods

### Variables and Data

#### Fetal Deaths

We use the fetal death public use data files (1996-2002) from the National Vital Statistics System, which compiles fetal death data from all fifty of the United States [21]. The National Center for Health Statistics (NCHS) defines a fetal death as a

"death prior to the complete expulsion or extraction from its mother of a product of human conception, irrespective of the duration of pregnancy and which is not an induced termination of pregnancy.

The death is indicated by the fact that after such expulsion or extraction, the fetus does not breathe or show any other evidence of life ..."

Most states require reporting of fetal deaths at or above 20 weeks of gestation. Although six states report fetal deaths before 20 weeks, 99% of these deaths have no information on sex. We therefore restricted our analysis to non-elective fetal deaths at or greater than 20 weeks.

Several quality assessments indicate underreporting of fetal deaths, especially those during the 20th to 27th weeks of gestation [22]. No research, however, suggests a sex bias in the reporting. Although several variables on the fetal death records frequently have missing values (e.g. prenatal care), fetal sex rarely has no value [21]. From January 1996 to December 2002, the NCHS imputed the sex of 4.65 percent of fetal deaths. To avoid potential misclassification of sex status, we excluded these deaths from the analysis.

#### Births

We acquired birth public use data files (1996-2002) from the National Vital Statistics System, which compiles birth certificate data from all registered births in the United States [23]. NCHS collaborates with state governments to provide access to information contained on all birth certificates in the U.S. NCHS makes these data available to the public; the data do not contain personal unique identifiers. Reporting of births in the U.S. is believed to be more than 99% complete [24].

### Analysis

We use interrupted time-series designs to test the hypotheses that the United States fetal death sex ratio rose following September 11, 2001. Researchers typically assume that, under the null hypothesis, the statistically expected value of a variable is its mean. The fetal death sex ratio, however, may exhibit secular trends, seasonal cycles, or the tendency to remain elevated or depressed after high or low values. These patterns, referred to collectively as autocorrelation, complicate hypothesis tests because the expected value of an autocorrelated series is not its mean.

Researchers have adjusted for autocorrelation by "decomposing" time series into predictable and residual components. This approach removes temporal patterns from the dependent variable before testing the effect of the independent variable and precludes spurious associations due to shared autocorrelation. Our analysis, therefore, is net of seasonality or other patterns in the sex ratio of fetal deaths.

We implemented the approach, recommended in the epidemiologic literature, through the following steps [25]. First, we used Auto Regressive, Integrated, Moving Average (i.e., ARIMA) methods to detect and model autocorrelation in the fetal death sex ratio from January 1996 through December 2002 [26]. The residuals of this model exhibit no autocorrelation and have an expected value of 0.

Second, we added a dichotomous variable scored 1 for September 2001 and 0 otherwise to the best fitting ARIMA models for the fetal death sex ratio. We specified the model such that we could measure the association between the fetal death sex ratio and September 11 for three monthly birth cohorts starting on September 2001 and ending November 2001.

Third, we estimated the equations resulting from step 2 and inspected their residuals to detect any autocorrelation. If any autocorrelation was detected, we added ARIMA parameters and estimated the resulting models. Fourth, we removed from the initial models any coefficients that did not reach conventional levels of statistical significance (p < .05).

## Results

### Fetal Death Sex Ratio

Over the test period, the mean numbers of reported monthly male and female fetal deaths in the U.S. (less California) were 995 and 871, respectively. The mean fetal death sex ratio was 1.14. As indicated in Table 1., non-Hispanic white mothers accounted for 49 percent of the fetal deaths, and over one-half of all deaths occurred to mothers with an education at or below high school.

**Table 1:** Descriptive characteristics of fetal deaths >20 weeks of gestation in the United States (less California), 1996-2002 (n = 156,510).

	N	%
Male sex	83,456	53.32
		
Maternal Age		
< 18 years	10,013	6.40
18-25 years	58,658	37.48
26-34 years	62,123	39.69
≥ 35 years	25,716	16.43
		
Maternal Education		
Less than high school graduate	30,823	19.69
High school graduate	50,812	32.47
Some college	26,806	17.13
College graduate	24,374	15.57
Unknown/not reported	23,695	15.14
		
Maternal Race/Ethnicity		
Non-hispanic white	76,316	48.76
Non-hispanic black	42,782	27.33
Hispanic	20,529	13.12
Other	16,883	10.79

The fetal death sex ratio series exhibited autocorrelation in that high or low values were followed twelve months later by smaller outlying values in the opposite direction. We removed this pattern (best modeled as an autoregressive process at a lag of 12 months, see Table 1) from the series before adding the binary September 11 variable.

Table 2 shows the results of the final model in which we add a constant, the September 11 variable, and any discovered autocorrelation. Consistent with the male fetal loss explanation, the U.S. fetal death sex ratio (less California) rose above its expected value in September 2001. Fetal death sex ratios in October and November 2001, however, did not differ significantly from expected values.

**Table 2:** Coefficients for model of the fetal death sex ratio (i.e., male/female) in the United States (less California) for September, October, and November 2001. Standard errors appear in parentheses.

Predictor	Initial model	Final model
Constant	1.143** (.0036)	1.143** (.0035)
		
ARIMA parameters		
AR at 12	-.4823** (.1132)	-.4713** (.1103)
AR at 3	-.2558* (.1188)	-.2657* (.1164)
		
September 11 variable lagged at:		
September 2001	.1279** (.0466)	.1232** (.0468)
October 2001	.0196 (.0463)	
November 2001	.0076 (.0462)	

* p < 0.05, 2-tailed test

** p < 0.01, 2-tailed test

We conducted several tests to determine if our result remained robust to control for potential analytic artifacts. To address whether outliers in the fetal death sex ratio affected the results, we applied outlier detection and correction routines to our original analyses [27]. The positive coefficient at September 2001 increased and remained statistically significant (coefficient = .1477, SE = .0377, p < .001, 1-tailed test). To ensure that non-constant variance of the series over time did not distort our estimates, we transformed the fetal death sex ratio to its natural logarithm and estimated the test equation again. Results, other than the metric of the September 2001 coefficient, remained essentially the same (.1111, SE = .0400, p < .01).

The month of September may exhibit unexpectedly high fetal death sex ratios even after we controlled autocorrelation at the twelfth month. We checked the robustness of our finding against this possibility by including as a control variable an indicator for all Septembers and re-estimating the equation. The coefficient for September 2001 remained positive and statistically significant (0.112, SE = .0552, p < .05).

We also examined whether results appear similar when we included all states in the analysis (i.e., adding California). As in the original test, the coefficient for September 2001 supports male fetal loss (0.103, SE = .047, p < .05) (full results available upon request).

### Secondary Sex Ratio

Support for the above hypothesis led us to test whether male fetal loss could also be detected *via *a fall in the secondary sex ratio two, three, or four months after the terrorist attacks. We applied the time series routines described above to the monthly secondary sex ratio series. Unlike the earlier test, we measure the association between September 11 and the secondary sex ratio starting on November 2001 and ending January 2002. We center the test on December 2001 since an excess of male fetal loss, particularly from 20 to 28 weeks of gestation, immediately after September 11 could precede a reduced odds of a male birth 12 weeks later (i.e., an estimated 32 to 40 weeks gestation). Research in California and New York City, moreover, reports lower than expected secondary sex ratios in December 2001 and January 2002, respectively.

The NCHS recorded 23,604,405 births in the U.S. (less California and New York City) over the seven-year test period. The mean monthly secondary sex ratio was 1.048. The secondary sex ratio series exhibited seasonality in that high or low values were followed six months later by smaller outlying values in the opposite direction. We removed this pattern (best modeled as an autoregressive process at a lag of 6 months, see Table 3) from the series. The ARIMA model also detected that unusually high or low values were "remembered" into the following month (modeled as an autoregressive process at a lag of 1 month).

**Table 3:** Coefficients for initial and final model of the secondary sex ratio in the United States (less California and New York City) for November 2001, December 2001 and January 2002.

Predictor	Initial model	Final model
Constant	1.048* (.0005)	1.048* (.0004)
		
ARIMA parameters		
AR at 1	.2403* (.1136)	.2487* (.1108)
AR at 6	-.2294* (.1128)	-.2353* (.1097)
MA at 16	.2986* (.1249)	.2933* (.1243)
		
September 11 variable lagged at:		
November 2001	-.0019 (.0038)	
December 2001	-.0079* (.0039)	-.0074* (.0036)
January 2002	-.00005 (.0038)	

Standard errors appear in parentheses.

* p < 0.05, 2-tailed test

Table 3 displays the results in which we add a constant, the September 11 variable, and any discovered autocorrelation. The secondary sex ratio in December 2001 fell below its expected value (coefficient = -.0079, SE = .0039, p < 0.05, 2-tailed test).

We tested whether outliers in the series may have inflated the variance [27]. No outliers were detected. We also transformed the secondary sex ratio to its natural logarithm to ensure that non-constant variance did not distort our findings. The coefficients did not appreciably change. We then tested the possibility that our findings arise from predictably low secondary sex ratios in all Decembers by adding an indicator variable for all Decembers and re-estimating the equation. The December 2001 coefficient remained negative and increased in magnitude (-.0107 SE = .004, p < .01).

We then included data from California and New York City to determine whether the secondary sex ratio in the entire U.S. fell after September 11. The results support a drop in the secondary sex ratio in December 2001 as the coefficient was negative and statistically significant (-.0069, SE = .0036, p < .05, 1-tailed test) (results available upon request).

## Discussion

Analysis of fetal deaths in the U.S. (less California) indicates that the terrorist attacks of September 11 coincided with an increase in the fetal death sex ratio in September 2001. Results converge with findings in California and support the communal bereavement hypothesis in that ambient shocks may affect the U.S. population of gravid mothers by threatening the gestation of male more than female fetuses [18].

The communal bereavement hypothesis contends that societies may react adversely to unsettling national events despite having no direct connection to persons involved in these events. This hypothesis builds on reports that witnessing harm to others triggers physiological responses in the witness that mirrors those in the persons harmed [28]. These responses can include the production of corticosteroids alluded to above as those implicated in the spontaneous abortion of males [29].

The literature reports that between 10 to 30 percent of fetal deaths greater than 20 weeks gestation are registered with state and federal vital statistics departments [30,31]. This under-registration indicates that using our discovered coefficient to estimate the total number of male fetuses lost in the U.S. as a result of September 11 would most likely approximate the lower bound of the true effect.

Strengths of our study include the population-based nature of NCHS data and coverage of over 156,000 fetal deaths over the test period. We have, in fact, tested our theory in the universe of exposed gestations. Use of this large population avoids instabilities in sex ratios associated with small numbers of fetal deaths. We also tested a population shock that assessments independent of ours established as stressful. The shock, moreover, affected the population at an unambiguous point in time allowing us to use the temporal patterning of responses to test male fetal loss. Results cannot arise from trends, seasonality, or other temporal patterns in the fetal death sex ratio because our methods removed autocorrelation.

Limitations of our test include the welcomed rarity of population stressors such as the events of September 11, 2001. Our findings may not generalize to other, more commonly experienced stressors on the population, although analyses in California suggest that regular swings in the economy may affect male fetal deaths [9]. In addition, we cannot know when in gestation (e.g., 20-24^th ^week, 24-28^th ^week, etc.) male fetuses appear most sensitive to population stressors. Whereas the NCHS gathers information on gestational age of the fetus, this variable often receives no score and has questionable accuracy even when reported [30]. Data limitations also preclude examination of physiologic mechanisms that may connect population stressors to male fetal loss. We await further research to identify these mechanisms.

Consistent with male fetal loss, the secondary sex ratio fell three months after the attacks. This finding indicates that an excess of males scheduled to be born in December 2001 may have been lost *in utero *due to the sequelae of the terrorist attacks. We also explored whether the secondary sex ratio in the entire U.S. fell below expected values eight, nine, and ten months after September 11, 2001. A decline in the secondary sex ratio in any of these months would support the reduced male conceptions argument. Findings provide no evidence for reduced male conceptions in that the secondary sex ratios eight, nine, and ten months after the terrorist attacks did not differ from expected values (results not shown).

A recent study of active-duty military families found no drop in the live birth sex ratio among mothers in their first trimester during September 11, 2001 [32]. We reconcile our findings with this null result in two ways. First, consistent with the literature that reports heightened male sensitivity to stressors after the 20^th ^week of gestation, we specified an induction period of 3 to 5 months (rather than 7 to 9 months) between the terrorist attacks and the birth sex ratio [6,7,18,19]. Second, our examination of the universe of gestations in the US may permit identification of sex ratio changes that would remain otherwise undetected when studying samples.

## Conclusions

Despite the fact that the fetal death rate after the 20th week of gestation (6.2 deaths per 1,000 live births and fetal deaths) approaches the infant mortality ratio (6.9 deaths per 1,000 live births), relatively little research and public health attention has focused on fetal death [31,33]. This circumstance arises in part from the lack of etiologic knowledge and perceived lack of opportunities for prevention. Here we report one population-based test that furthers understanding of psychosocial antecedents of fetal death. Results, moreover, suggest that the fetal death sex ratio may serve as a sentinel indicator of the degree to which pregnant women, at least, react to population stressors.

## Competing interests

The authors declare that they have no competing interests.

## Authors' contributions

TAB co-designed the research question, retrieved the data, performed the statistical analysis, and served as the lead author of the manuscript. RC co-designed the research question, assisted with the statistical analysis, and co-authored the Methods and Discussion. JA assisted with the research design, performed the data management, assisted with interpretation of the data, and edited all sections. All authors read and approved the final manuscript.

## Pre-publication history

The pre-publication history for this paper can be accessed here:

http://www.biomedcentral.com/1471-2458/10/273/prepub
